# Degradation Mechanism of Concrete Subjected to External Sulfate Attack: Comparison of Different Curing Conditions

**DOI:** 10.3390/ma13143179

**Published:** 2020-07-16

**Authors:** Gaowen Zhao, Mei Shi, Mengzhen Guo, Henghui Fan

**Affiliations:** 1 School of Highway, Chang’an University, Xi’an 710064, China; yubi_shangguan@163.com; 2 Department of Geotechnical Engineering, Tongji University, Shanghai 200092, China; 3 College of Natural Resources and Environment, Northwest A&F University, Yangling 712100, China; 4 College of Water Conservancy and Architectural Engineering, Northwest A&F University, Yangling 712100, China; yt07@nwsuaf.edu.cn

**Keywords:** cast-in-situ concrete, degradation mechanism, sulfate attack, sulfate diffusion

## Abstract

Sulfate induced degradation of concrete brings great damage to concrete structures in saline or offshore areas. The degradation mechanism of cast-in-situ concrete still remains unclear. This paper investigates the degradation process and corresponding mechanism of cast-in-situ concrete when immersed in sulfate-rich corrosive environments. Concrete samples with different curing conditions were prepared and immersed in sulfate solutions for 12 months to simulate the corrosion of precast and cast-in-situ concrete structures, respectively. Tests regarding the changes of physical, chemical, and mechanical properties of concrete samples were conducted and recorded continuously during the immersion. Micro-structural and mineral methods were performed to analyze the changes of concrete samples after immersion. Results indicate that the corrosion process of cast-in-situ concrete is much faster than the degradation of precast concrete. Chemical attack is the main cause of degradation for both precast and cast-in-situ concrete. Concrete in the environment with higher sulfate concentration suffers more severe degradation. The water/cement ratio has a significant influence on the durability of concrete. A lower water/cement ratio results in obviously better resistance against sulfate attack for both precast and cast-in-situ concrete.

## 1. Introduction

Sulfates, chlorides, and other corrosive sources are widely observed in soils, ground water, river water, and seawater in salty saline and offshore areas [1,2,3,4,5]. Among the mostly observed ions in the environment, sulfates have been claimed to be one of the most aggressive ions to bring great damage to concrete structures [6,7,8,9,10,11].

Experimental and theoretical studies regarding the degradation of concrete caused by sulfate attack have been well reported in recent decades. The corrosion products such as ettringite, gypsum, or thaumasite were reported after sulfate corrosion [12,13]. In addition, researchers found that hydrates of sulfates, especially mirabilite, were responsible for the development of cracks in concrete in sulfate-rich environments [14,15]. Meanwhile, some researchers proposed that both chemical and physical attack induced by sulfate presence should be considered, which directly lead to the degradation of concrete, since both chemical corrosion products and crystallization hydrates have been detected in the damaged concrete [16]. Therefore, it is widely accepted that the degradation of concrete induced by sulfate attack can be divided into physical sulfate attack, chemical sulfate attack, and physical–chemical combined degradation [17,18].

Concrete subjected to sulfate attack always results in great expansion as well as the risk of failure [19,20,21]. The expansion is reported to be caused by the corrosion products or hydrates induced during sulfate attack [22,23]. It has been reported that the volume of chemical corrosion products, especially ettringite, far surpasses the volume of reactants during chemical corrosion [24,25,26]. In addition, the strength of corrosion products is relatively low, leading to a great decrease of concrete strength [27]. It is notable that the volume of hydrates, Na_2_SO_4_·10H_2_O, is several times greater than the volume of Na_2_SO_4_ [28,29,30]. Therefore, chemical or physical attack by sulfates has a significant negative effect on the performance of concrete [31].

It is clear that previous studies regarding the degradation of concrete induced by sulfate attack are of great importance for further research. By paying close attention to the published research, most of the previous studies were conducted on precast concrete samples. However, it should be pointed out that most concrete structures in salty saline or offshore areas are cast-in-situ concrete structures, especially for underground concrete structures. It should be noted that the construction processes of precast and cast-in-situ concrete structures are quite different. For cast-in-situ concrete, the corrosion process starts immediately after the casting of fresh concrete in the sulfate-rich environments, while for precast concrete structures, the hardening process has finished before being installed in the environment. Moreover, the effects of the water/cement ratio on the sulfate induced degradation of cast-in-situ concrete are rarely reported, which plays an important role in affecting the properties and safety of cast-in-situ structures. Consequently, the degradation process and mechanism between the precast and cast-in-situ concrete are quite different. Moreover, the safety of cast-in-situ concrete cannot be ensured if the design of cast-in-situ concrete structures is based on the research conducted on precast concrete samples. Thus, it is necessary to perform a specific experiment to reveal the degradation process and mechanism differences between the precast and cast-in-situ concrete.

To study the degradation process and mechanism of cast-in-situ concrete caused by sulfate attack, concrete samples in cylinder shapes were prepared and immersed in sulfate solutions. Different curing conditions were used in the present study. To highlight the differences of specimens with different curing conditions, specimens cured for 12 h were described as cast-in-situ concrete and specimens cured for 28 days were described as precast concrete in the present study. Tests were conducted to record the physical, chemical, and mechanical properties changes during the 12-month immersion. The micro-structural and mineral properties of concrete were analyzed after immersion by X-ray diffraction (XRD), energy dispersive X-ray spectroscopy (EDS), and scanning electron microscopy (SEM) tests.

## 2. Materials and Methods

### 2.1. Materials

To prepare concrete specimens, P.C. 32.5 R Portland cement (Conch, Shanghai, China) was used in the present experiment [32,33]. The chemical composition of cement is presented in Table 1. River sand was used in this study as fine aggregate. The maximum particle size was 2.36 mm, and the fineness modulus was 2.6. Artificial crushed stone was used as coarse aggregate. Excessively large aggregate in concrete would result in great discreteness of the test results due to the size of specimens, thus the maximum size of crushed stone was 10 mm. Distilled water was used to prepare concrete samples and sulfate corrosive solutions.

To investigate the effects of water/cement ratio on the degradation of concrete caused by sulfate attack, three kinds of concrete samples were designed in the present study. Mixture proportions of concrete samples in the present study are listed in Table 2.

### 2.2. Sample Preparation and Testing Methods

#### 2.2.1. Sample and Solution Preparation

Na_2_SO_4_ was selected to prepare sulfate corrosive solutions. The sulfate concentrations of different solutions were set at 0%, 3%, 5%, and 10% (named as CK, 3S, 5S, and 10S, as shown in Table 3) in the present study. It should be noted that the sulfate concentration and liquid depth of each solution were checked every month to ensure that the sulfate concentration was constant and all the specimens were fully immersed in the solutions. The details of specimens and solutions are presented in Table 3. It should be emphasized that to get a relatively accurate value of concrete properties, three parallel tested samples were prepared simultaneously in different tests in the present study.

Raw materials, such as river sand, crushed stone, and cement, were weighed and mixed in the mixer before the distilled water was added. Freshly mixed concrete was then put in the modules, and the size of concrete specimens was 100 mm × 200 mm (diameter × height). Samples with modules were then put in the curing chamber with a temperature of 22 °C and relative humidity of >95%. The top and bottom surfaces of each specimen were sealed with epoxy resin. For precast concrete, the samples were cured for 28 days before being put into the solutions, while for cast-in-situ concrete, the samples were put into the solutions after being cured for 12 h.

#### 2.2.2. Physical Properties

It should be noted that the specimens were put into an oven at a temperature of 60 °C for 48 h after being immersed in the solutions for 1, 3, 6, 9, and 12 months. To determine the diameter and weight changes of concrete samples during the immersion, a Vernier caliper and an electronic scale were selected to measure the diameter and weight of specimens. The diameter of each specimen was measured 5 times near the mid-height of specimens to obtain an average diameter. The weight of each specimen was weighed twice, and the average value was recorded.

#### 2.2.3. Compressive Strength

An unconfined compressive strength test was performed to determine the compressive strength of each specimen during the immersion. A loading system (the equipment model was POPWIL (HSR)) with a maximum capacity of 1000 kN was used to conduct the compressive strength test, and the loading rate during the tests was set as 5 kN/s.

#### 2.2.4. Sulfate Concentration

To determine the sulfate concentration in the concrete specimens, a drill was used to collect the powder samples by drilling perpendicular to the lateral surface of each specimen. Five powder samples (from the surface to a depth of 25 mm beneath the exposed surface of specimens) were obtained and sealed in the plastic zip bags. Each powder sample was mixed with 50 mL distilled water and put into a bottle for 48 h. Then the solution was filtered through filter paper to remove residues. The sulfate concentration of each solution was then determined by standard chemical titration [34].

#### 2.2.5. Micro-Structural and Mineral Analyses

SEM (by ZEISS-SIGMA 300 system, Carl Zeiss (Shanghai), Shanghai, China) and XRD (by model RINT 2000 X-ray diffractometer, Rigku (Beijing), Beijing, China) analyses were performed to observe the micro-structures and to determine the mineral composition of concrete samples after being immersed in sulfate solutions for 12 months. The element analysis (by EDS (ZEISS-SIGMA 300, Carl Zeiss (Shanghai), Shanghai, China)) was also performed in the SEM test to determine the corrosion products. The samples used in the SEM analysis were concrete core samples drilled from the lateral surface of specimens with a depth of 10 mm beneath the exposed surface. The powder samples used in the XRD analysis were drilled from the exposed surface with a depth of 5 mm and passed through a 75-µm sieve.

## 3. Results and Discussion

It is well accepted that the micro-structural and mineral properties play an important role in affecting the basic physical and mechanical properties of concrete structures. Therefore, the results of the micro-structural and mineral analyses were given and discussed firstly. Additionally, sulfates penetrate concrete and react with concrete compositions to generate corrosion products and hence change the properties of concrete. Thus, the diffusion process of sulfates into concrete is subsequently introduced. Finally, results of other properties are given and discussed.

### 3.1. Micro-Structural and Mineral Analyses

**Figure 1:**
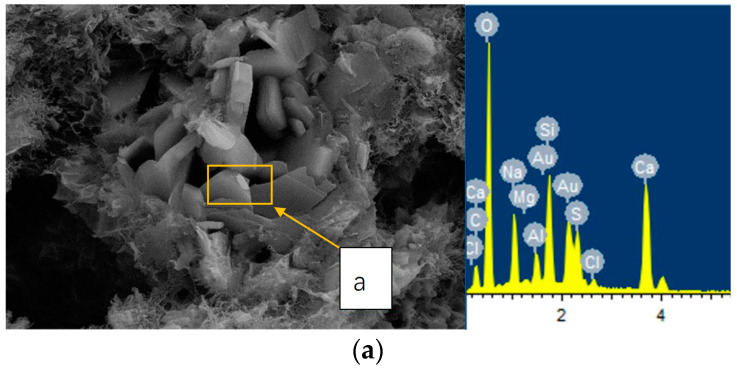

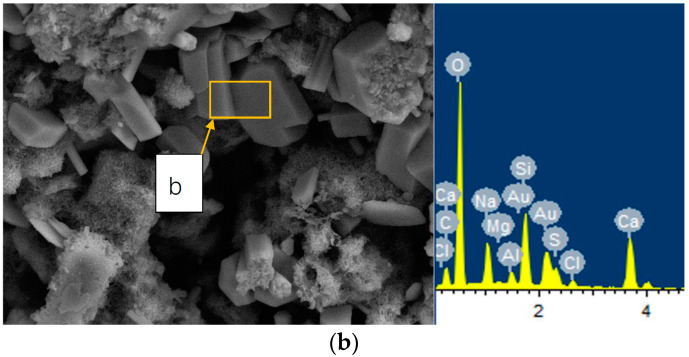
SEM images and corresponding EDS results in concrete exposed to 10% sulfate solutions for (**a**) precast concrete, magnification = 5 K, and (**b**) cast-in-situ concrete, magnification = 2.5 K.

**Figure 2:**
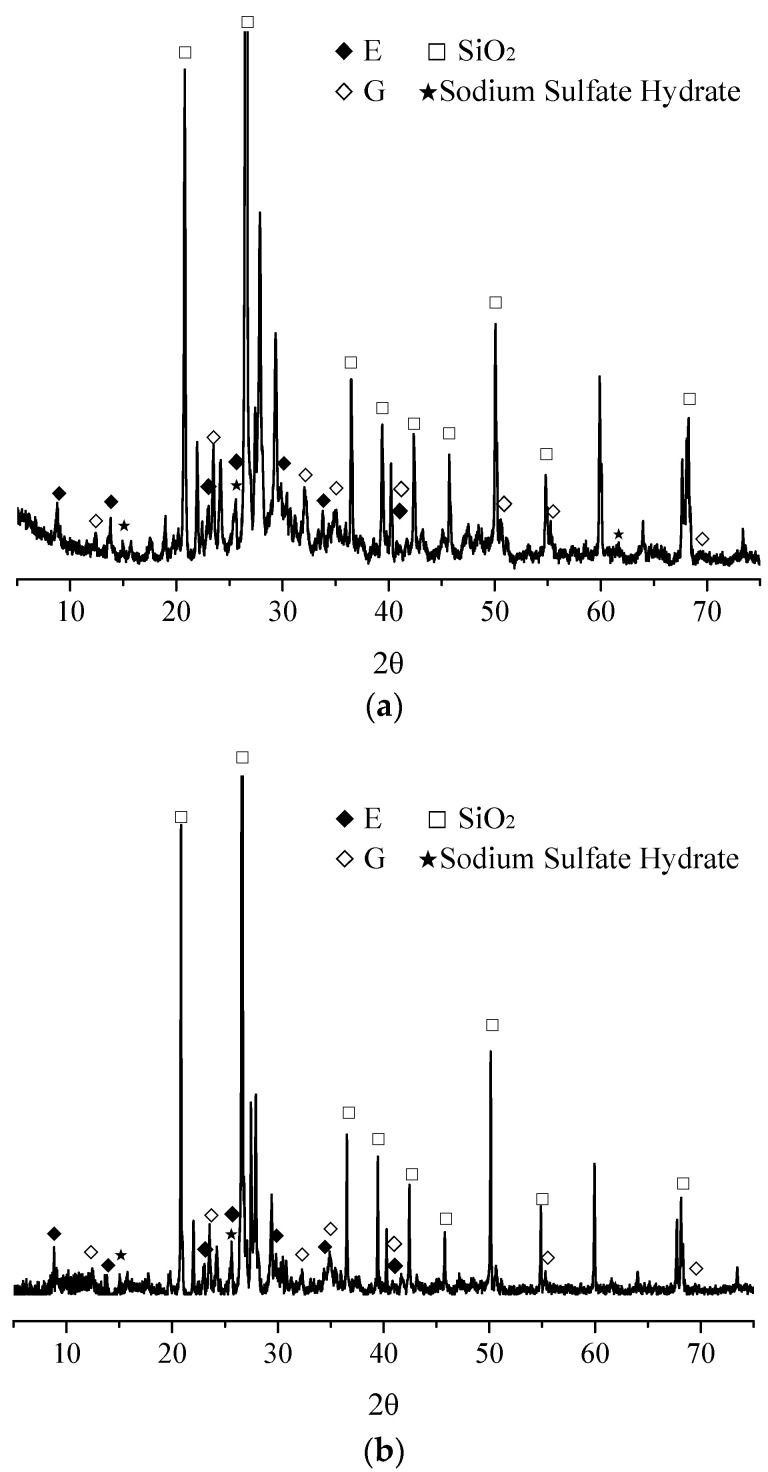
XRD analysis results of corrosion products in the concrete for (**a**) cast-in-situ concrete, and (**b**) precast concrete.

To show the micro-structures of concrete after immersion in sulfate solutions for 12 months, SEM observation was conducted, and the results are presented in Figure 1. SEM images in Figure 1a,b were taken from precast concrete and cast-in-situ concrete specimens after immersion in 10% sulfate solution for 12 months, with the same water/cement ratio of 0.55. To give a relatively solid determination of the corrosion products after immersion. XRD tests were performed on different concrete samples after immersion, and the results are shown in Figure 2. The EDS analysis results are also listed on the right side of the SEM images to give reliable proof. The results of XRD analysis in Figure 2a,b were obtained from the precast and cast-in-situ concrete specimens immersed in 10% sulfate solution, and the water/cement ratios were 0.55.

It is clear in the SEM images and EDS analysis results that sulfate corrosion products such as ettringite and gypsum were observed in both precast and cast-in-situ concrete samples. It should be noted that the peaks of Au and S shared little overlap in the EDS results, and a and b in the SEM images in Figure 1 were the sampling points of EDS analysis. The analysis results of XRD tests also indicated that the peaks of corrosion products of ettringite (marked as E in XRD patterns), gypsum (marked as G in XRD patterns), and sulfate hydrates were detected in the concrete after immersion in sulfate solutions. The involved chemical corrosion reactions of sulfate attack can be summarized as follows [35,36,37]:

(1) $${2\mathrm{Na}}^{+}+{{\mathrm{SO}}_{4}}^{2-}+10H_{2}O\Leftrightarrow {\mathrm{Na}}_{2}{\mathrm{SO}}_{4}\cdot 10H_{2}O$$

(2) $$3\mathrm{CaO}\cdot {\mathrm{Al}}_{2}O_{3}+3\left({\mathrm{CaSO}}_{4}\cdot 2H_{2}O\right)+26H_{2}O\to 3\mathrm{CaO}\cdot {\mathrm{Al}}_{2}O_{3}\cdot 3{\mathrm{CaSO}}_{4}\cdot 32H_{2}O$$

(3) $$3\mathrm{CaO}\cdot {\mathrm{Al}}_{2}O_{3}\cdot {\mathrm{CaSO}}_{4}\cdot 12H_{2}O+2\left({\mathrm{CaSO}}_{4}\cdot 2H_{2}O\right)+16H_{2}O\to 3\mathrm{CaO}\cdot {\mathrm{Al}}_{2}O_{3}\cdot 3{\mathrm{CaSO}}_{4}\cdot 32H_{2}O$$

(4) $$4\mathrm{CaO}\cdot {\mathrm{Al}}_{2}O_{3}\cdot 13H_{2}O+3\left({\mathrm{CaSO}}_{4}\cdot 2H_{2}O\right)+14H_{2}O\to 3\mathrm{CaO}\cdot {\mathrm{Al}}_{2}O_{3}\cdot 3{\mathrm{CaSO}}_{4}\cdot 32H_{2}O+\mathrm{CaO}\cdot H_{2}O$$

(5) $${\mathrm{Ca}(\mathrm{OH})}_{2}{+\mathrm{Na}}_{2}{\mathrm{SO}}_{4}{+2H}_{2}O\to {\mathrm{CaSO}}_{4}\cdot {2H}_{2}O+2\mathrm{NaOH}$$

The results of micro-structural and mineral analyses illustrate that the main corrosion products in both precast and cast-in-situ concrete were the same. Thus, one can conclude that both precast and cast-in-situ concrete mainly suffer chemical sulfate attack, especially when the concrete is fully immersed in the sulfate-rich environments. The peaks of sulfate hydrates detected in concrete indicated that crystallization of sulfates also occurred in the cracks and pores of concrete. However, it should be emphasized that fully immersed concrete samples stayed beneath the liquid surface all the time, and the evaporation process was not involved during the immersion. Thus, it was inferred that the crystallization hydrates of sulfates were generated during the 48-hours drying process instead of the immersion period.

The generation of sulfate corrosion products would generally affect the properties of concrete in the following three ways. Firstly, the corrosion products would be generated and embedded in concrete, affecting the subsequent development of other properties. The reason is that the properties of sulfate-induced corrosion products and concrete are quite not the same. Secondly, the generated corrosion products would be gradually accumulated in the initial cracks and pores in concrete, influencing the density and strength of the outer layer of concrete. At last, it is well accepted that the corrosion products would cause great volume expansion by loading great pressure on the walls of internal cracks and pores, putting the outer layer of concrete under great tensile stress.

### 3.2. Sulfate Concentration

**Figure 3:**
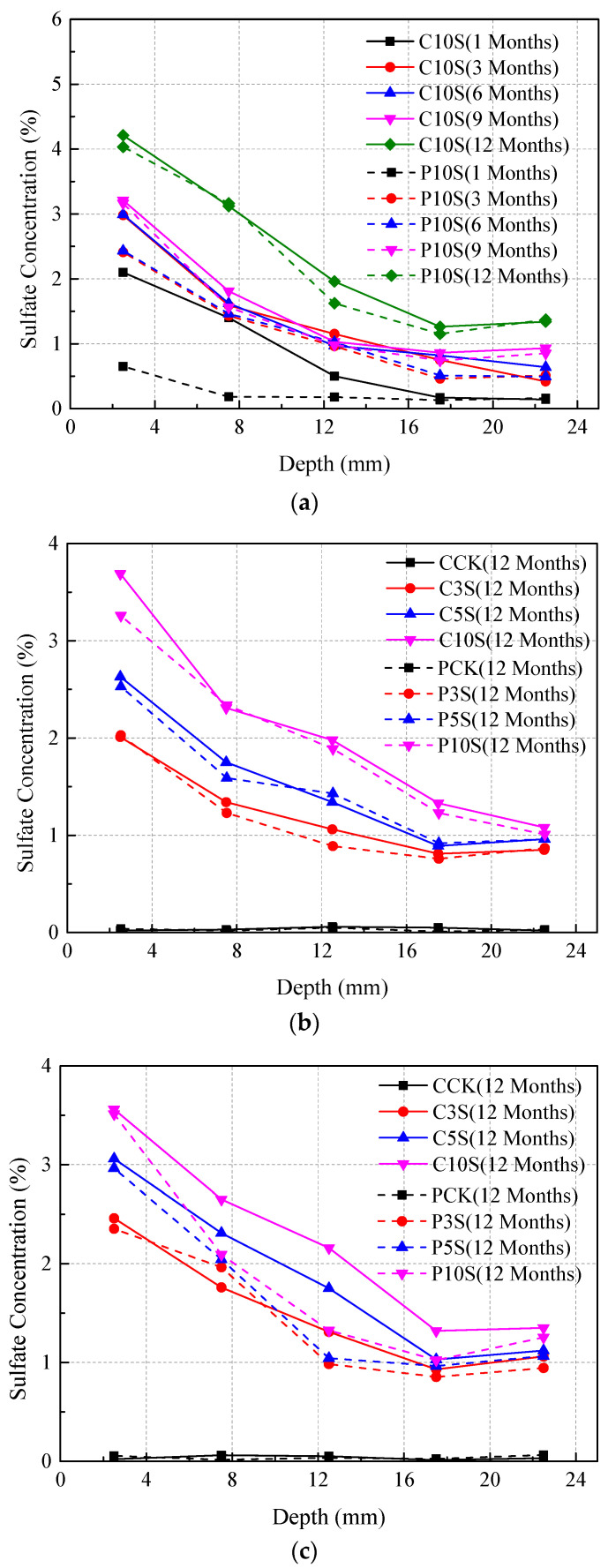

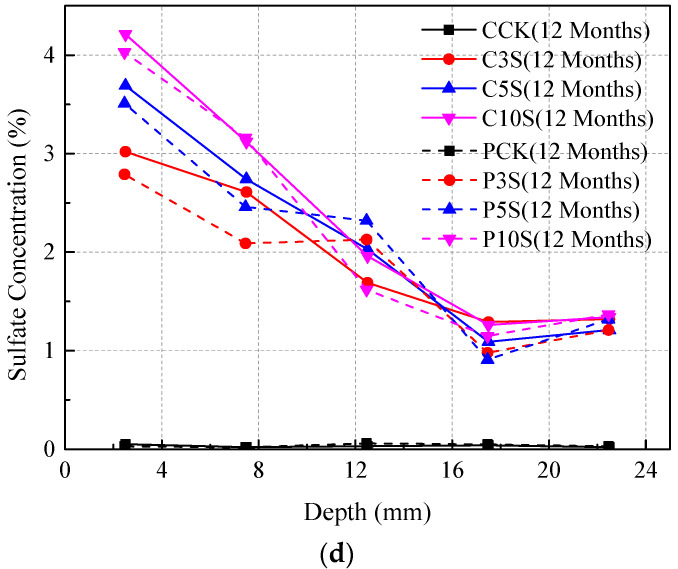
Sulfate concentration of concrete with different water/cement ratios: (**a**) 0.55, in 10% sulfate solution, (**b**) 0.40, in different solutions, (**c**) 0.48, in different solutions, and (**d**) 0.55, in different solutions.

Concentration of sulfates in different concrete samples was determined, and the results are shown in Figure 3. Overall, the results clearly indicated that the concentration of sulfates was relatively high near the surface, and then decreased fast with the growth of depth beneath the exposed surface of concrete specimens.

For precast concrete specimens, it is clear that the penetration of sulfate ions was relatively slow, especially for concrete immersed in 3% sulfate solution. The concentration of sulfates in concrete immersed in 5% and 10% sulfate solutions was higher and the penetration depth was deeper. Moreover, it is clear that the sulfate concentration in concrete with lower water/cement ratio is lower than that in concrete with greater water/cement ratio.

As for cast-in-situ concrete, it was noticed that the sulfate concentration was relatively higher than that in precast concrete specimens. In addition, sulfates reached deeper depth in cast-in-situ concrete. Thus, sulfates diffused faster in cast-in-situ concrete, especially for concrete specimens with high water/cement ratio.

The diffusion of sulfates into concrete can be divided into two steps. In the first step, the soluble sulfates such as Na_2_SO_4_ dissolve in water to form a sulfate solution in which sulfate ions can move freely. In the second step, sulfate ions would move from positions with higher sulfate concentrations to positions with lower sulfate concentrations. In this regard, the diffusion of sulfate ions is mainly motived by the concentration gradient between the concrete and the outer environments. In addition, it should be emphasized that the porosity of concrete also plays an important role in affecting the diffusion of sulfates. It is obvious that concrete with higher porosity can physically provide more paths for the penetration of sulfates. Thus, the sulfate concentration in concrete with a higher water/cement ratio is relatively high, while on the contrary, sulfates only reach a shallower depth beneath the exposed surface of concrete with a lower water/cement ratio. 

It should be noted that the diffusion of sulfates in precast concrete and cast-in-situ concrete is different. First of all, sulfate ions can diffuse directly into concrete once the fresh concrete is put into the environment during the hardening process. Then, the penetrated sulfate ions can react with the cement composition to generate sulfate corrosion products such as gypsum and ettringite. The embedded corrosion products affect the integrity of concrete and hence increase the initial porosity of concrete. Additionally, the strength of sulfate induced corrosion products is relatively low, making it easier for expansive products to generate more cracks in the upcoming stage. Thus, the diffusion of sulfates in cast-in-situ concrete is easier and faster than that in precast concrete structures. 

It is clear that the porosity of concrete with different water/cement ratios is different. The porosity is lower when the water/cement ratio is lower, resulting in better waterproofness. Thus, it would be harder for an external sulfate solution to permeate into the internal concrete areas and hence decreases the diffusion speed of sulfate ions. Therefore, the sulfate concentration is low and the penetration depth is shallow in concrete with a lower water/cement ratio. Furthermore, concrete with a lower water/cement ratio has a higher tensile strength, making it difficult for sulfate corrosion products to generate new cracks, while for concrete with a high water/cement ratio, it would be easier for expansive products to extend initial cracks or generate new cracks, leading to a faster degradation.

### 3.3. Physical Properties

**Figure 4:**
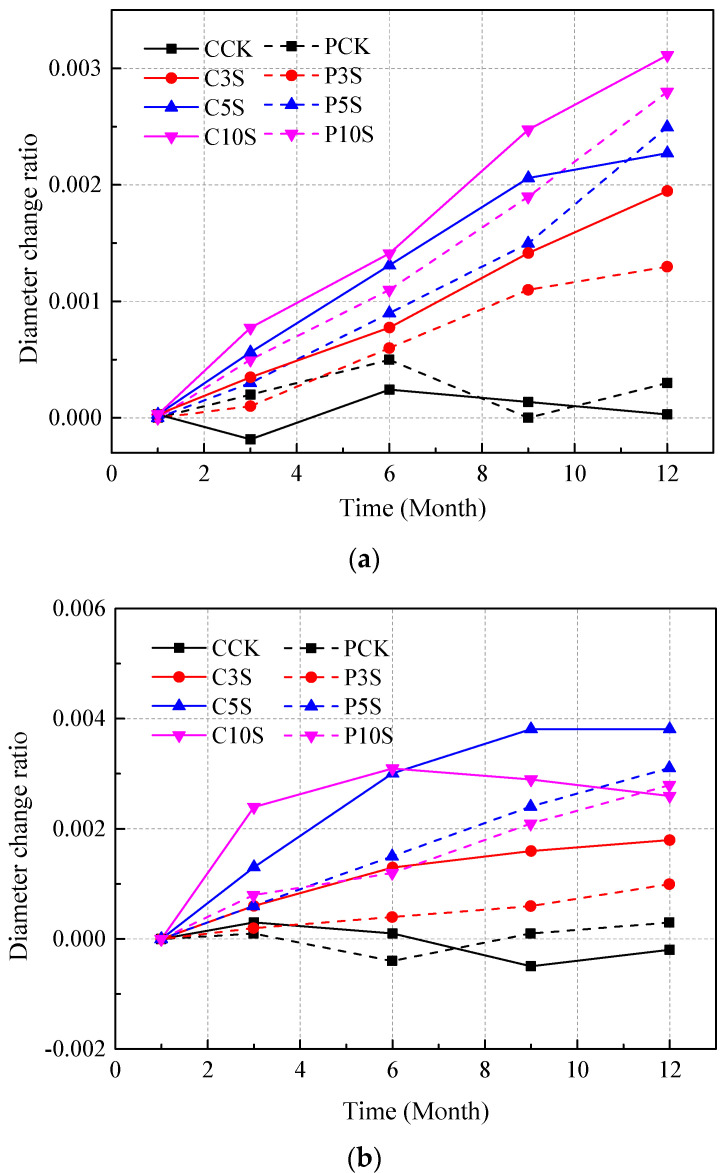

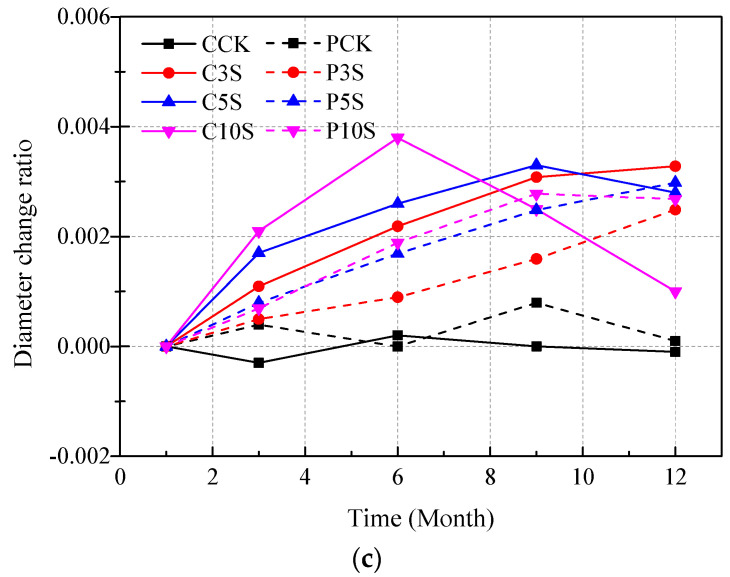
Diameter change ratios for concrete with different water/cement ratios: (**a**) 0.40, (**b**) 0.48, and (**c**) 0.55.

The diameter and weight of different specimens in different solutions were measured and recorded during the immersion. To give a clear comparison of diameter for different concrete specimens, the change ratios of diameter were calculated by

(6) $$a=\frac{d_{t}-d_{0}}{d_{0}}\times 100\%$$

in which *a* is the change ratio, *d_0_* is the initial diameter, and *d_t_* is the diameter of the specimen after a specific immersion period of *t*. The results are shown in Figure 4. 

The comparison clearly indicates that concrete in sulfate solutions suffered radical expansion. For precast concrete specimens, the diameter showed a relatively stable increase tendency during the immersion. Only the specimen immersed in 10% sulfate solution showed a relatively slight decrease after the ninth month (with a water/cement ratio of 0.55). Additionally, one could observe that concrete in solution with a higher concentration of sulfates suffered more radical expansion in the early stage, indicating that concrete suffered more serious degradation when immersed in the environments with a relatively higher sulfate concentration. 

Compared to precast concrete specimens, it can be seen in Figure 4 that cast-in-situ concrete obviously encountered more volume changes during the immersion. As for concrete immersion in 5% and 10% solutions, the radius increased quickly in the early corrosion stage but soon began to decrease even after the sixth month. Moreover, one could observe visible cracks on the surface of cast-in-situ concrete specimens. The spalling and shedding of concrete powder and fragments were also observed on the surface of concrete in a 10% sulfate solution.

**Figure 5:**
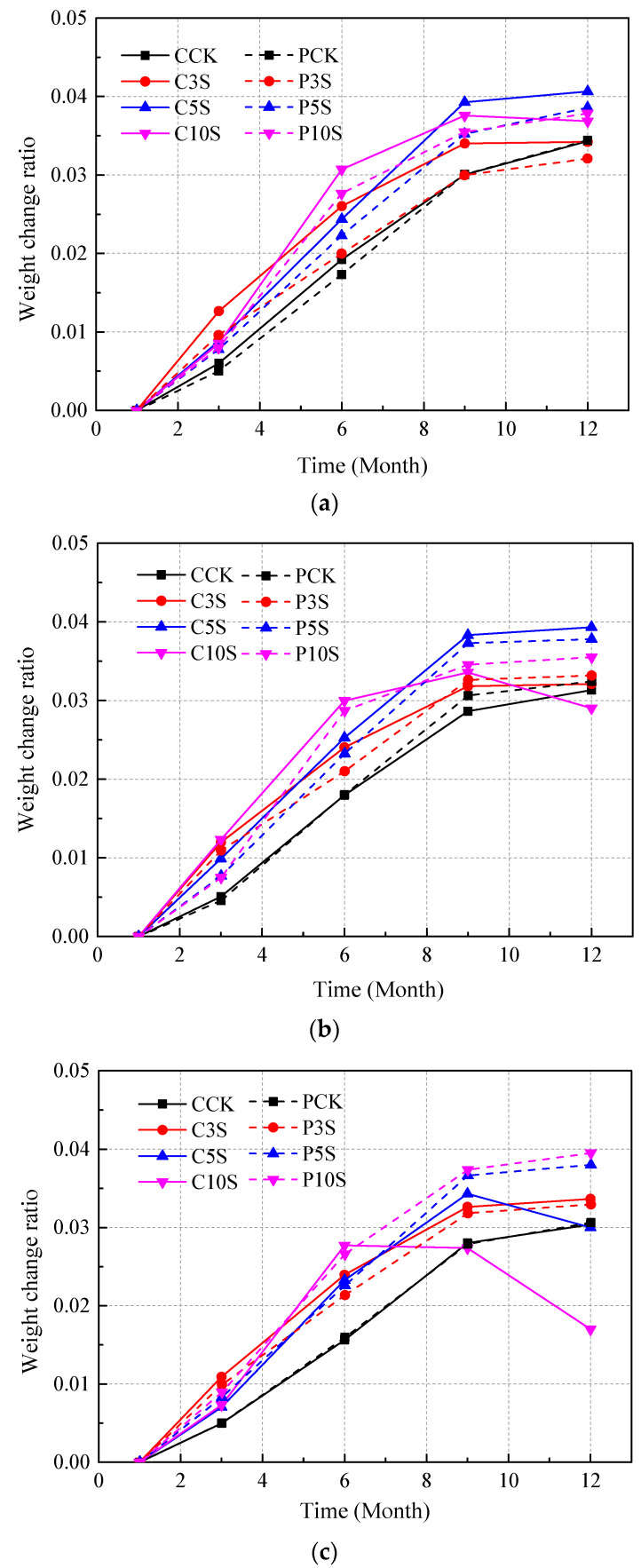
Weight change ratios for concrete with different water/cement ratios: (**a**) 0.40, (**b**) 0.48, and (**c**) 0.55.

For precast concrete samples, the weight increased quickly in the early corrosion stage and became relatively stable after nine months. Furthermore, there was a slight difference between the concrete in distilled water and sulfate solutions, which was caused by the accumulation of sulfate corrosion products. In addition, it can be seen that concrete immersed in solutions with higher sulfate concentration obviously experienced more fluctuation during the immersion, and the weight increment was relatively greater, especially at the early stage. However, it can be seen that the weight of cast-in-situ concrete experienced more significant changes during the 12-month immersion. The weight of specimens increased quickly at the early stage, then became stable or even began to decrease after the sixth month, especially for specimens immersed in 10% sulfate solution (shown in Figure 5c).

**Figure 6:**
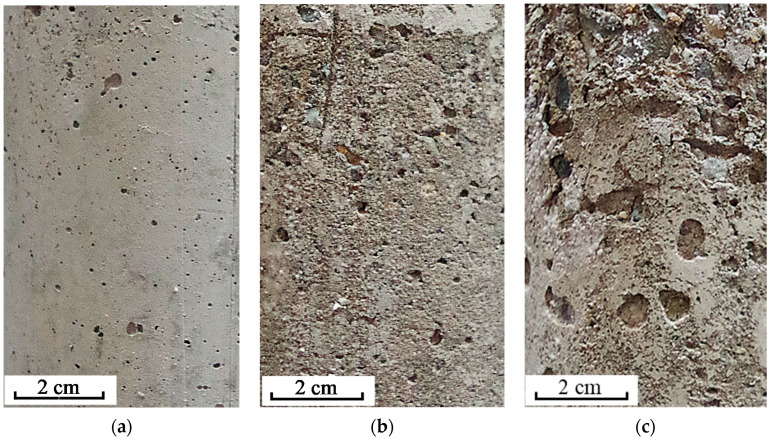
Surface photos of specimens with different water/cement ratios: (**a**) 0.40, (**b**) 0.48, and (**c**) 0.55.

To give a clear comparison of degradation between the precast and cast-in-situ concrete, the photos of the exposed surface of specimens after immersion are shown together. The image in Figure 6a shows the exposed surface of the precast concrete with a water/cement ratio of 0.48 immersed in a 10% sulfate solution. The images in Figure 6b,c show the exposed surfaces of the cast-in-situ concrete immersed in 10% sulfate solutions, while the water/cement ratios are 0.48 and 0.55, respectively. The comparison distinctly shows that more potential cracks were generated on the exposed surface of cast-in-situ concrete. Moreover, there was clearly more shedding of concrete powder and fragments on the cast-in-situ concrete surface. The appearance comparison illustrates that precast concrete has a better resistance performance against sulfate attack than that of cast-in-situ concrete. Additionally, the results illustrate that concrete with a low water/cement ratio clearly suffers less degradation and shows a better performance against sulfate attack compared to the sample with a high water/cement ratio.

It is clear that the diameter and weight of concrete samples are significantly affected by sulfate attack. In the early corrosion stage, sulfates penetrates internal concrete and reacts with concrete composition to generate ettringite and gypsum. The corrosion products are accumulated in the initial cracks and pores with immersion time. Soon after, the initial cracks are fully filled by the corrosion products, and excessive pressure is loaded on the walls of cracks and pores to cause volume expansion. During this stage, the diameter and weight of specimens increase with immersion time, while in the later corrosion stage, the excessive filling of corrosion products loads great pressure on the walls of the crack, causing the extending of initial cracks and generation of new cracks. Moreover, the development of a crack system on the concrete surface causes serious spalling and shedding of concrete powder and fragments, which cause a decrease of both the diameter and weight of concrete specimens. Therefore, the diameter of concrete specimens is a comprehensive result of the increase tendency induced by the proceeding of expansion and the decrease tendency induced by the falling off of concrete on the exposed surface. The weight of concrete specimens is a comprehensive result of the increase tendency induced by the accumulation of sulfate corrosion products and the decrease tendency induced by the shedding of concrete fragments. Furthermore, the diameter and weight of specimens are mainly controlled by the increase tendency in the early corrosion stage, while in the later stage, the decrease tendency plays the leading role. Hence both the diameter and weight of concrete specimens encounter a rapid decrease at the end of the immersion, especially for concrete with a high water/cement ratio immersed in the solution with a high sulfate concentration (such as the specimen C10S shown in Figure 4c and Figure 5c). From the results, it can be inferred that cast-in-situ concrete suffered more severe degradation in the later corrosion stage than precast concrete. In addition, the spalling and shedding of concrete fragments became more serious at the later stage, which damaged the integrity of the outer layer of concrete and then caused potential loss of concrete strength.

### 3.4. Mechanical Properties

**Figure 7:**
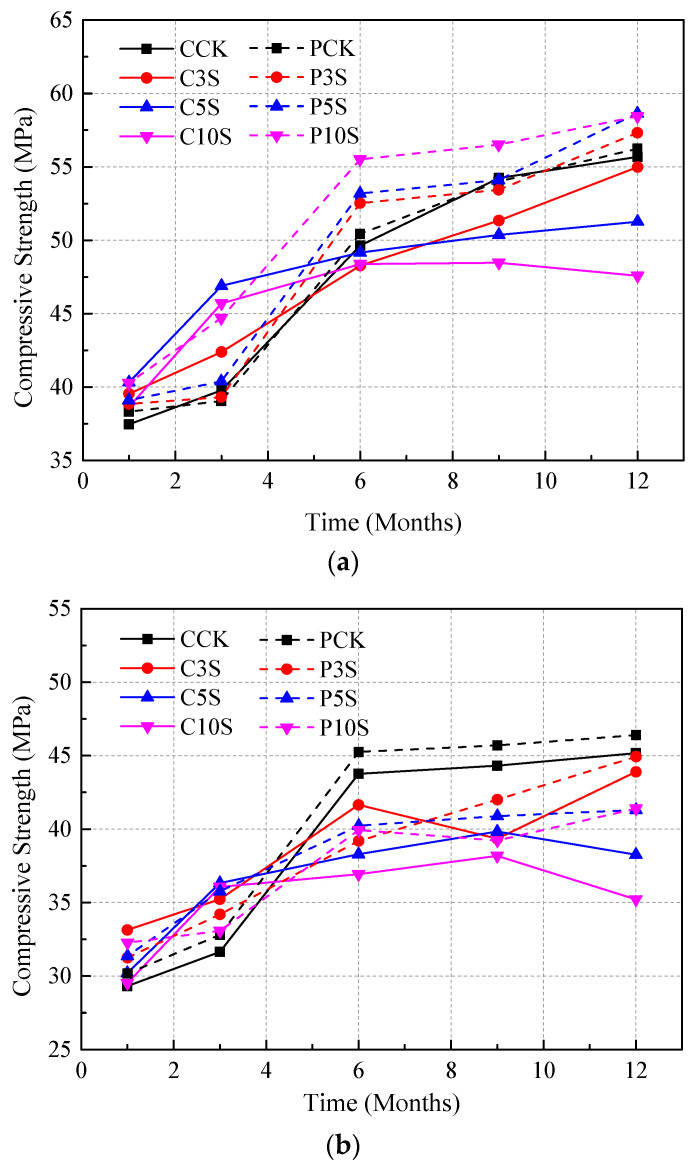

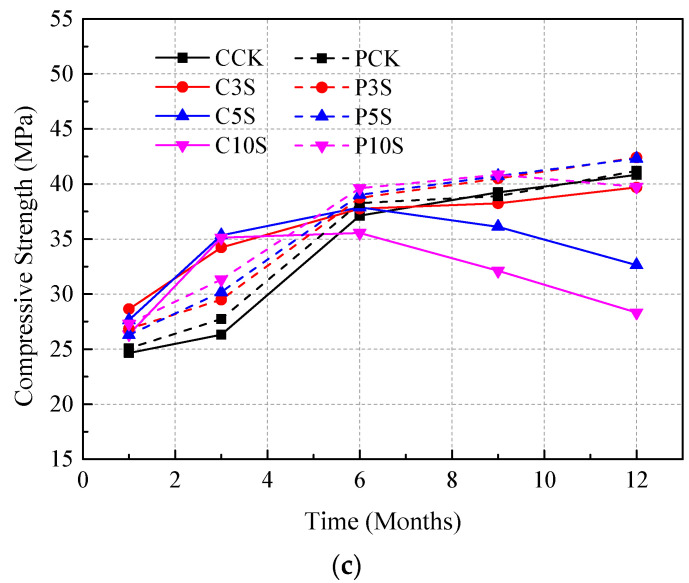
Compressive strength of concrete with different water/cement ratios: (**a**) 0.40, (**b**) 0.48, and (**c**) 0.55.

The results of compressive strength tests are presented in Figure 7. It is clear that the strength of different specimens in different solutions is quite different.

For precast concrete specimens, the strength showed a relatively stable increase tendency during the immersion, especially for concrete specimens immersed in 3% and 5% sulfate solutions. For concrete immersed in 10% sulfate solution (with water/cement ratio of 0.55), the strength began to decrease after the ninth month, while for concrete specimens with water/cement ratios of 0.40 and 0.48, the strength remained increasing or stable even at the end of the immersion.

It is noteworthy that the strength of cast-in-situ concrete increased quickly, and the value was relatively low in the early stage, while after that, the increase tendency of strength became stable or even began to decrease after the sixth month. In addition, one could notice that concrete lost more strength when immersed in the solutions with higher sulfate concentration.

It is clearly illustrated in Figure 7 that the water/cement ratio had a significant influence on the strength of concrete specimens. Generally, the strength of concrete with a greater water/cement ratio was clearly lower than the strength of concrete with a lower water/cement ratio. Moreover, the results distinctly indicated that the strength of concrete with a higher water/cement ratio began to decrease earlier, and the strength was relatively low at the end of the immersion. In addition, the strength of concrete in the solution with a higher sulfate concentration decreased faster and lost more strength after immersion.

For precast concrete specimens, the hardening process and strength development were finished before being put into the sulfate solutions. Thus, sulfate ions had to penetrate internal concrete from the outside-in with immersion time. Sulfate ions penetrated the outer layer of concrete together with the permeating of pore water during the immersion. Then, the penetrated sulfate ions reacted with the concrete composition to generate corrosion products, which accumulated in the initial cracks and pores to cause expansion and degradation. Therefore, the degradation of precast concrete proceeded from the surface into the internal areas of the concrete gradually. However, for cast-in-situ concrete, the strength development of concrete was negatively affected in the early corrosion stage, since the hardening process of concrete was not complete during this stage. Hence, the strength of cast-in-situ concrete was lower than that of precast concrete, even when the specimens were immersed in the same sulfate solutions, especially when the sulfate concentration was high. 

It has to be emphasized that the water/cement ratio has a significant effect on the strength and resistance performance of concrete against sulfate attack. It is well accepted that concrete with a lower water/cement ratio always results in a relatively higher density and lower porosity. Therefore, the strength of specimens with lower water/cement ratio is relatively high. In addition, the porosity of concrete also plays a significant role in affecting the diffusion speed of sulfate ions, subsequently influencing the mechanical property of concrete. 

The generation and accumulation of sulfate corrosion products in the cracks and pores influence the properties of concrete. In the very early corrosion stage, the cracks and pores are not fully filled; hence, the accumulation of corrosion products do not affect the strength of concrete. Then, the excessive filling of corrosion products generate pressure on the walls of internal cracks and pores, making the concrete denser and causing a slight increase in strength. Then, initial cracks are extended and new cracks are generated, decreasing the strength of concrete to a large extent. In this regard, for concrete with a higher water/cement ratio, it takes less time for sulfates to penetrate internal concrete regions and brings a great deal of damage to concrete structures. Moreover, the tensile strength of the internal concrete area with a higher water/cement ratio is low, making it easier for sulfate corrosion products to generate new cracks and bring great damage.

## 4. Conclusions

Experiments were performed to investigate the degradation of precast and cast-in-situ concrete caused by sulfate attack. Parameters regarding physical, chemical, and mechanical properties were determined and compared in detail. Methods of SEM, EDS, and XRD were used to reveal the micro-structural and mineral changes after immersion. Based on the experiments in the present study, the main conclusions are as follows:

Degradation of both precast and cast-in-situ concrete is caused by chemical sulfate attack when the concrete is fully immersed in the sulfate-rich environments. The main corrosion products induced by sulfate attack are ettringite and gypsum.

Sulfate attack results in great expansion and weight loss in the later corrosion stage, putting concrete structures in great danger, especially for cast-in-situ concrete structures. The diffusion of sulfates is faster in cast-in-situ concrete than that in the precast concrete due to the faster development of cracks in the cast-in-situ concrete.

The faster strength increase of cast-in-situ concrete in the early corrosion stage is induced by the faster accumulation of corrosion products. Cast-in-situ concrete obviously suffers more strength loss during corrosion. Precast concrete has better resistance performance against sulfate attack than cast-in-situ concrete structures, indicating that precast concrete structures are more suitable in sulfate-rich environments.

## Figures and Tables

**Table 1:** Chemical components of cement.

Chemical Components	Al_2_O_3_	SO_3_	Fe_2_O_3_	MgO	CaO
Content (%)	6.82	1.96	2.13	1.92	60.05

**Table 2:** Mixture proportions of concrete in the present study.

w/c	Concrete Components			
	Water (kg/m^3^)	Cement (kg/m^3^)	Sand (kg/m^3^)	Gravel (kg/m^3^)
0.40	162	405	600	1275
0.48	175	365	638	1238
0.55	189	345	685	1210

Note: w/c is the water/cement ratio.

**Table 3:** The details of specimens and solutions.

Specimens	Immersion Solution	Specimens	Immersion Solution
PCK	Distilled water	CCK	Distilled water
P3S	3% Na_2_SO_4_ solution	C3S	3% Na_2_SO_4_ solution
P5S	5% Na_2_SO_4_ solution	C5S	5% Na_2_SO_4_ solution
P10S	10% Na_2_SO_4_ solution	C10S	10% Na_2_SO_4_ solution

Note: P for precast concrete specimens and C for cast-in-situ concrete specimens.
